# An Engineered Microvirin Variant with Identical Structural Domains Potently Inhibits Human Immunodeficiency Virus and Hepatitis C Virus Cellular Entry

**DOI:** 10.3390/v12020199

**Published:** 2020-02-11

**Authors:** Munazza Shahid, Amina Qadir, Jaewon Yang, Izaz Ahmad, Hina Zahid, Shaper Mirza, Marc P. Windisch, Syed Shahzad-ul-Hussan

**Affiliations:** 1 Department of Biology, Syed Babar Ali School of Science and Engineering, Lahore University of Management Sciences, Lahore 54792, Pakistan; 14130004@lums.edu.pk (M.S.); 16140029@lums.edu.pk (A.Q.); 18140018@lums.edu.pk (I.A.); 15140003@lums.edu.pk (H.Z.); shaper.mirza@lums.edu.pk (S.M.); 2 Applied Molecular Virology Laboratory, Discovery Biology Division, Institut Pasteur Korea, 696, Seongnam 13488, Korea; jaewon.yang@ip-korea.org (J.Y.); marc.windisch@ip-korea.org (M.P.W.); 3 Division of Bio-Medical Science and Technology, University of Science and Technology, Daejeon 34141, Korea

**Keywords:** microvirin, lectin, human immunodeficiency virus, hepatitis C virus, antiviral inhibitor, non-immunogenic, viral entry, protein drugs, LUMS1

## Abstract

Microvirin (MVN) is one of the human immunodeficiency virus (HIV-1) entry inhibitor lectins, which consists of two structural domains sharing 35% sequence identity and contrary to many other antiviral lectins, it exists as a monomer. In this study, we engineered an MVN variant, LUMS1, consisting of two domains with 100% sequence identity, thereby reducing the chemical heterogeneity, which is a major factor in eliciting immunogenicity. We determined carbohydrate binding of LUMS1 through NMR chemical shift perturbation and tested its anti-HIV activity in single-round infectivity assay and its anti-hepatitis C virus (HCV) activity in three different assays including HCVcc, HCVpp, and replicon assays. We further investigated the effect of LUMS1 on the activation of T helper (T_h_) and B cells through flow cytometry. LUMS1 showed binding to α(1-2)mannobiose, the minimum glycan epitope of MVN, potently inhibited HIV-1 and HCV with EC_50_ of 37.2 and 45.3 nM, respectively, and showed negligible cytotoxicity with CC_50_ > 10 µM against PBMCs, Huh-7.5 and HepG2 cells, and 4.9 µM against TZM-bl cells. LUMS1 did not activate T_h_ cells, and its stimulatory effect on B cells was markedly less as compared to MVN. Together, with these effects, LUMS1 represents a potential candidate for the development of antiviral therapies.

## 1. Introduction

Human immunodeficiency virus (HIV-1) and hepatitis C virus (HCV) infections continue to be a healthcare challenge globally, accounting for an enormous disease burden [1,2,3]. An effective vaccine against these viruses remains to be developed. Within the past decade, advancement in the development of anti-viral regimens has improved the situation, particularly in controlling HCV infections [4,5]. However, the outcome of antiviral therapies could be limited by several factors, including the possible emergence of drug-resistant viral variants. This scenario, therefore, signifies the continuous efforts towards the development of new anti-viral therapies or preventive measures.

The common feature between HIV-1 and HCV is the presence of highly glycosylated outer envelope—the envelope glycoprotein 120 (gp120) of HIV-1 and E2 of HCV exhibits over 20 and 11 *N*-glycosylation sites, respectively [6,7,8]. This glycan shield decorating the surface of viruses has been exploited as a potential target for therapeutic or preventive interventions. In recent years, carbohydrate-binding agents, in particular, lectins have been identified which can inhibit cellular entry of the viruses by specifically binding to these viral surface glycans [9,10,11,12]. Given their potent nature, use of these lectins as topical microbicides has been suggested for the prevention of sexual transmission of HIV-1 [13,14,15]. As an example, griffithsin, a lectin from red algae, is currently in phase-1 clinical trials [16]. Two of the major challenges hampering the clinical application of these lectins are their potential cytotoxicity and immunogenicity. In general, chemical and structural heterogeneity of proteins is one of the primary factors responsible for their immunogenicity.

**Figure 1:**
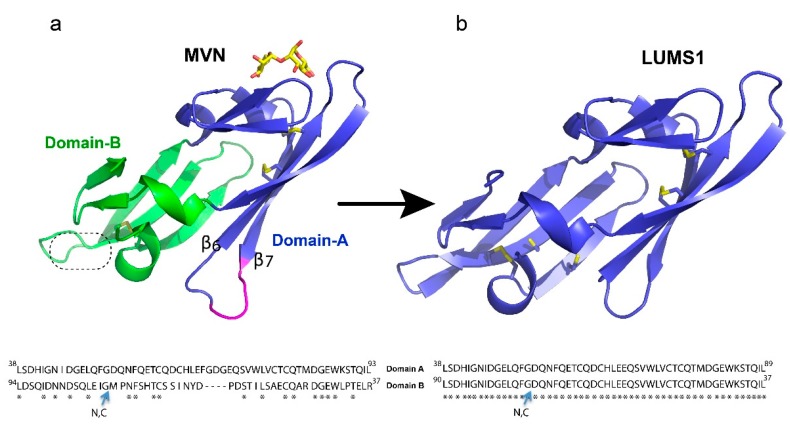
Description of the protein design: (**a**) microvirin (MVN) structure (PDB ID 2YHH) shown in cartoon presentation with two structural domains colored blue and green while bound glycan is colored yellow. Insertion of four amino acids in domain-A as compared to domain-B is indicated in magenta. The second putative carbohydrate binding site is indicated by a dotted circle. (**b**) The homology-modeled structure of LUMS1 was created through SWISS-MODEL online tools using MVN as a template. Qualitative model energy analysis (QMEAN) scoring function was used to access the quality of the model. Side chains of all cysteine residues in both proteins are shown in gold sticks. Alignment of amino acid sequence of two domains of MVN and LUMS1 is shown at the bottom of the respective protein structure. N, C indicates N- and C-termini of the protein sequences.

Microvirin (MVN) is one of the potent anti-HIV lectins, which was initially isolated from *Microcystis aeruginosa* and has been shown to have only minor cytotoxicity and mitogenic effects as compared to other antiviral lectins [17,18]. MVN has been reported to specifically recognize α(1-2)mannobiose present at the termini of branched high mannose type glycans on the viral surface. This 12 kDa lectin consists of two structural domains, which share 35% sequence identity, and unlike other anti-viral lectins, it exists as a monomer (Figure 1a). Moreover, there is a four residues long insertion in domain-A as compared to domain-B of MVN [19]. In this study, we engineered an MVN variant, LUMS1 (the name derived from Lahore University of Management Sciences), exhibiting 100% sequence identity between its two structural domains, thereby markedly decreasing the chemical heterogeneity. We investigated this protein for its potential to inhibit cellular entry of HIV and HCV, and studied its cytotoxicity, carbohydrate specificity, and preliminary effects on the activation of immune cell surface markers.

## 2. Materials and Methods

### 2.1. Protein Expression

For the recombinant expression of LUMS1, the gene encoding for LUMS1 amino acid sequence was synthesized through commercial facilities (Genscript, Piscataway, NJ, USA), sub-cloned into pET32a expression vector, subsequently expressed in a bacterial system (BL21 strain), and purified through different chromatographic techniques including nickel-affinity, size exclusion, and ion exchange chromatography. For the expression of the ^15^N-labelled protein, the transformed bacteria were grown in minimal media supplemented with ^15^N-ammonium chloride as the only source of nitrogen. The purified protein was transferred into PBS buffer of pH 7.4 for all biological assays, and into 20 mM phosphate buffer containing 50 mM NaCl for NMR experiments, through dialyses using dialysis membrane of 3.5 KDa cutoff (Slide-A-Lyzer™ MINI Dialysis Device, Thermo Fisher Scientific, Waltham, MA, USA) [19].

### 2.2. NMR Experiments

NMR experiments were performed on Bruker Avance Neo 600 MHz NMR spectrometer equipped with TXI triple resonance probe at 298 K. Two dimensional ^15^NHSQC spectra were recorded with 16 scans and 256 data points in the indirect dimension. Topspin 4.0.5 software was used to acquire and process the NMR data [19].

### 2.3. HIV Inhibition Assay

HIV-1 entry inhibition by LUMS1 was studied by using pseud-typed virus-based single-round infectivity assay, according to a previously reported method [20]. In this regard, LUMS1 at varying concentration was mixed with HXB2 strain of HIV-1 pseudo-typed viral particles at 37 °C followed by the addition of TZM-bl cells (NIH AIDS reagent program) at a concentration of 1 × 10^4^ cells/100 µL. After 48 h, cells were lysed and percent infection was measured through luciferase activity (BrightGlo, Promega, Maddison, WI, USA) for each dilution of inhibitor with respect to control containing no inhibitor. Similarly, the activity of LUMS1 against vesicular stomatitis virus (VSV) was also tested using virus pseudo-typed with VSV envelope and HIV-1 backbone.

### 2.4. HCV Infection Assay

The anti-HCV activity of LUMS1 was evaluated using cell culture-derived infectious HCV (HCVcc) expressing an NS5A-GFP fusion protein in the presence of inhibitors as previously described [21]. Briefly, Huh-7.5 cells were seeded in 384-well plates (2.5 ×  10^3^ cells/well). LUMS1 were serially diluted in complete DMEM, added to each well of the plates, inoculated with HCVcc and incubated at 37 °C for 3 days. On day 3 post-infection (p.i.), cultured cells were fixed with 2% paraformaldehyde in PBS containing 10 μg/mL Hoechst 33,342 (Life Technologies, Waltham, MA, USA) for 30 min. HCV replication was analyzed by determining the number of GFP-positive cells using automated confocal microscopy (Opera, PerkinElmer, Waltham, MA, USA). Cytotoxicity was assessed by counting cell nuclei stained with Hoechst 33342 and normalized to untreated control cells.

### 2.5. HCV Replication Assay

HCV subgenomic replicon cells were treated with inhibitors as described previously [21]. Briefly, replicon cells were seeded in 384-well plates and treated with inhibitors for 72  h. The inhibitory effect of the protein on HCV RNA replication was monitored together with RNA polymerase inhibitor (sofosbuvir) as a reference compound. Viral replication and cytotoxicity was assessed as described above.

### 2.6. HCV Pseudoparticle Assay

Viral entry was assessed using HCV pseudoparticle (HCVpp) expressing a luciferase reporter gene as described before [22]. In brief, HEK293 T cells seeded for 1 day in T-75 flasks were co-transfected with 5 μg of an HCV E1/E2 envelope protein expression vector (genotype 1a) and 15 μg of pNL4.3.Luc.R^–^ E^–^, HIV Gag-Pol expression packaging vector containing luciferase reporter gene using Lipofectamine 3000 (Invitrogen, Waltham, MA, USA). Supernatants containing the pseudoparticles were harvested at 72  h and used as HCVpp in the entry inhibition assay using Huh7.5 cells as described above in Section 2.3. EI-1, a known potent inhibitor of HCV entry [23] was used as a positive control at a single concentration of 10 μM.

### 2.7. Flow Cytometry Analysis of PBMCs

For the flow cytometry analysis blood from two healthy volunteers was collected after informed consent and PBMCs were isolated separately using density gradient centrifugation (Polymorphprep; Cosmo Bio, Tokyo, Japan) and washed with PBS (1% FBS). After treating PBMCs (10^6^ cells/mL) with varying concentrations of LUMS1, MVN, and cyanovirin-N (CVN) for 72 h at 37 °C and 5% CO_2_, cells were washed with PBS (2% FBS) and incubated with APC-conjugated anti-cluster of differentiation-4 (CD4), PE-conjugated anti-CD25, and percp cy5.5-conjugated anti-CD20 antibodies for 30 min at 4 °C. Respective isotype controls were used for compensation of backgrounds. Finally, cells were washed with PBS (2% FBS), fixed with 1% formaldehyde, and analyzed by FACS (Calibur; BD Biosciences, Franklin lakes, NJ, USA), using CellQuest software for data acquisition [18].

### 2.8. MTT Assay

TZM-bl cells and PBMCs were seeded in 96-well plates at optimized concentrations of 8 × 10^4^ and 7 × 10^5^ cells per 100 µL, respectively, with various concentrations of the LUMS1 protein (1.25, 2.5, 5, and 10 µM), and incubated for 48 h. MTT reagent was added at a final concentration of 0.5 mg/mL and incubated for 4 h as reported previously [24]. The plate containing PBMCs was centrifuged for 5 min at 300× *g*. After completely removing the media, 100 µL DMSO was added and thoroughly mixed to dissolve the crystals, and finally absorbance was measured at 570 nm.

## 3. Results

### 3.1. Designing of the LUMS1 Protein and Characterization of Its Carbohydrate Binding

A characteristic feature of HIV-1 entry inhibitor lectins is multivalent recognition through more than one carbohydrate-binding site to attain high avidity of interaction required for potent antiviral activity. MVN, however, contains one carbohydrate-binding site present in its domain-A (Figure 1a). In this design, we removed a four-residues long insertion between strands B6 and B7 in domain-A of MVN and changed the amino acid sequence of domain-B making it identical to that of domain-A and creating two carbohydrate-binding sites. The removal of the four residues corresponding to a long flexible loop could further minimize chemical heterogeneity and reduce the protein size. Since the domain-B of MVN has been reported to adopt the structure homologous to its domain-A without this insert [19], it was conceivable that the removal of these four residues may not disturb the protein folding. Subsequently, we built a homology model of the designed protein, LUMS1, using MVN as a template to obtain a preliminary idea about its structure. The resultant model exhibited a similar structure to MVN, but unlike MVN, each of the structural domains of LUMS1 contained a putative carbohydrate-binding site and two potential inter-strand disulfide linkages (Figure 1b). In order to experimentally investigate LUMS1, we produced the recombinant protein in two different forms, unlabeled and isotopically labeled with ^15^N.

**Figure 2:**
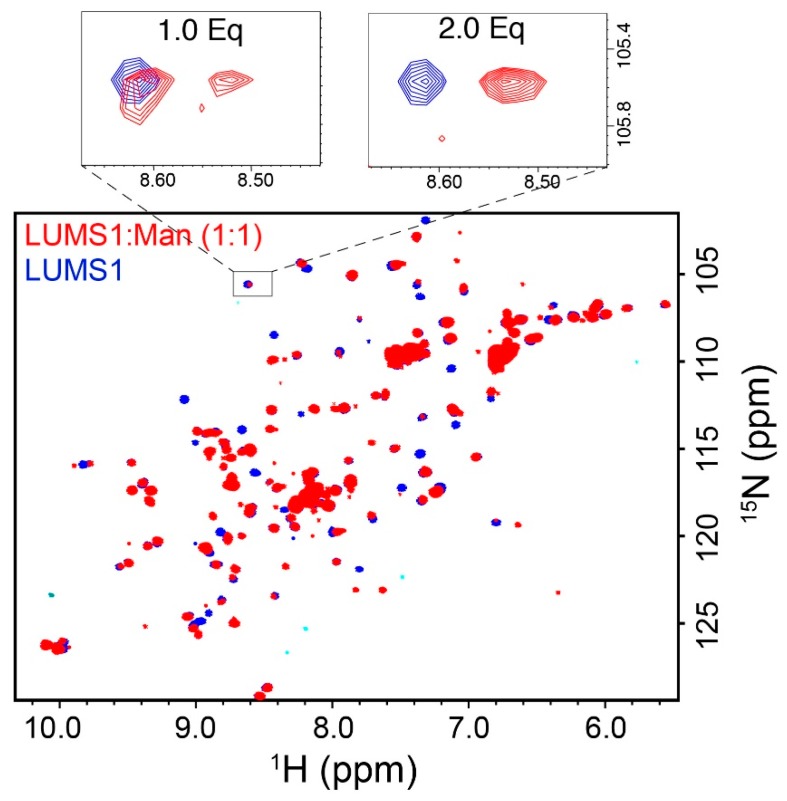
Carbohydrate binding of LUMS1: ^15^NHSQC spectra of LUMS1 alone (blue) and in the presence of one equivalent of the α(1-2)mannobiose glycan (red), superimposed. Expansions of a region of spectrum containing single cross-peak in the absence (blue) and presence of one and two equivalents of α(1-2)mannobiose are shown at the top.

To find out if the purified protein was folded, we recorded a two-dimensional ^15^NHSQC spectrum of isotopically labeled LUMS1. Well-dispersed ^1^H-^15^N correlation cross-peaks were observed indicating that protein was folded (Figure 2). In order to test the binding of LUMS1 to α(1-2)mannobiose, the minimum glycan epitope of MVN, we used the NMR chemical shift perturbation technique, as backbone ^1^H-^15^N resonances are sensitive to change in the environment resulting from the binding of a ligand [25]. In this regard, ^15^NHSC spectra were acquired on a sample containing ^15^N-lableled LUMS1 alone in solution and in the presence of increasing concentrations of α(1-2)mannobiose. On addition of α(1-2)mannobiose, cross-peaks of several amino acids in ^1^H-^15^N correlation spectra either broadened or underwent chemical shift changes, indicating the binding of the carbohydrate. Upon the addition of the carbohydrate at two equivalents of the protein molar concentration, titration appeared to be completed as no further changes in the spectra were observed upon addition of more quantities of the carbohydrate. ^15^NHSQC spectra of LUMS1 free and in the presence of one equivalent of α(1-2)mannobiose is shown in Figure 2 with the expansion of a cross-peak showing a stepwise change in chemical shift with the addition of one and two equivalents of carbohydrate. At one equivalent of carbohydrate, the cross-peak in the expanded region representing free and carbohydrate-bound state of LUMS1 appeared, while in the presence of two equivalents of carbohydrate, the cross-peak representing the free state of LUMS1 completely disappeared. This indicated the binding stoichiometry as 2:1 and the presence of two carbohydrate-binding sites on LUMS1.

### 3.2. LUMS1 Inhibits HIV-1 Cellular Entry

**Figure 3:**
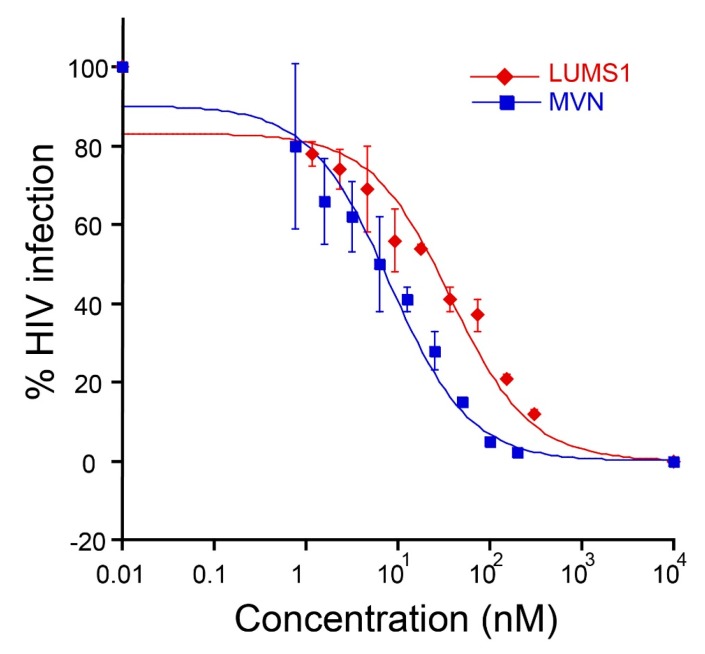
Human immunodeficiency virus (HIV-1) entry inhibition by LUMS1: dose-response curve showing inhibition of pseudo-typed virus, HIV-1 strain HXB2, by LUMS1 and MVN. The proteins at varying concentration was mixed with the virus at 37 °C followed by the addition of TZM-bl. After 48 h, cells were lysed, and percent infection was measured through luciferase activity. The assay was performed in triplicates.

We tested LUMS1 for its anti-HIV activity in pseud-typed virus-based single-round infection assay using the HXB2 strain of HIV-1 [19]. LUMS1 potently inhibited the HIV-1 entry with EC_50_ of 37.2 ± 4.4 nM (Figure 3) measured from its dose-response curve. For comparison, the activity of MVN was also determined as positive control, and its EC_50_ was measured as 8.0 ± 1.4 nM. To determine viral specificity, the activity of LUMS1 against an amphotropic virus, VSV, was tested and it was found that LUMS1 did not inhibit VSV at a concentration as high as 10 μM (Figure S2, Supplementary Material).

### 3.3. LUMS1 Inhibits HCV Cellular Entry

**Figure 4:**
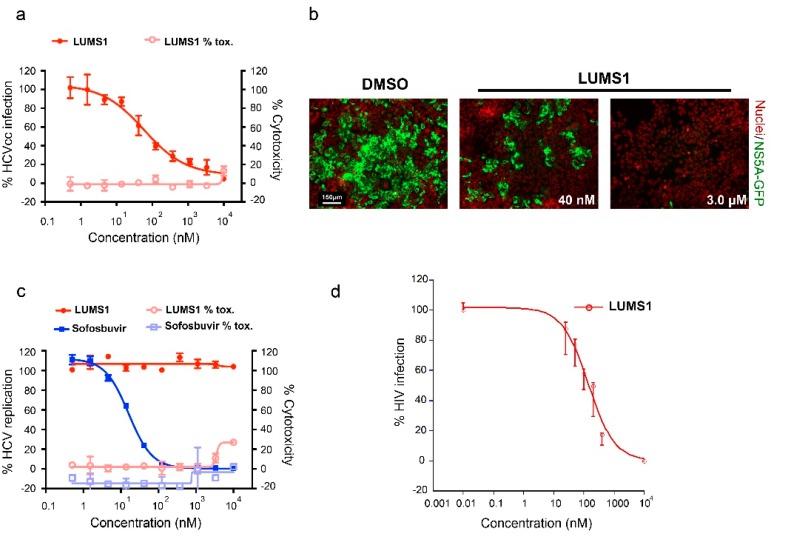
Hepatitis C virus (HCV) inhibition by LUMS1: (**a**) Anti-HCV activity in Huh-7.5 cells. Cells were pretreated with increasing concentrations of LUMS1 for 2 h followed by infection with HCVcc (JFH1) for 72 h in the presence of proteins. (**b**) HCV infectivity and total cell number were assessed by determining the number of GFP-positive cells (green) and nuclei (red), respectively, for 3 days in the presence of LUMS1). Images were acquired by confocal microscopy. (**c**) HCV subgenomic replicon cells were treated with LUMS1 and sofosbuvir. (**d**) HCVpp was mixed with different concentrations of LUMS1 and subsequently added to Huh-7.5 cells. After 72 h incubation, cells were lysed and percent infection was measured through luciferase activity (BrightGlo, Promega, USA) for each dilution of inhibitor with respect to control containing no inhibitor.

Next, to evaluate the effect of LUMS1 on HCV infection, we used cell culture-derived infectious HCV (HCVcc) expressing an NS5A-GFP fusion protein [21]. In this assay, the infection of liver-derived cell line Huh-7.5 by HCVcc was analyzed by determining the number of GFP-positive cells using automated confocal microscopy. The expression of the NS5A-GFP fusion protein, which served as a marker for productive HCV infection, was inhibited by LUMS1 in a dose-dependent manner with a calculated EC_50_ of 45.3 ± 18.6 nM and CC_50_ > 10 μM (Figure 4a). Representative images are shown in Figure 4b. To investigate whether LUMS1 can also interfere with some post entry event of viral replication cycle, we tested the effect of LUMS1 using subgenomic HCV replicon cells [21]. We observed that HCV replication was not affected by LUMS1 at a concentration as high as 10 μM, while it was significantly inhibited by sofosbuvir, a known inhibitor of HCV replication (Figure 4c). This suggested that LUMS1 interfered with HCV entry rather than HCV replication. Results of the HCVcc and replicon assays are summarized in Table 1. To further confirm that LUMS1 interferes with HCV E1/E2-mediated viral entry, the HCV pseudoparticle (HCVpp) system was employed [22]. LUMS1 inhibited the HCVpp with EC_50_ of 142.1 ± 23 nM demonstrating that LUMS1 specifically inhibited viral entry (Figure 4d).

### 3.4. LUMS1 Does Not Stimulate Cellular Activation Markers and Shows Negligible Cytotoxic Effect

**Figure 5:**
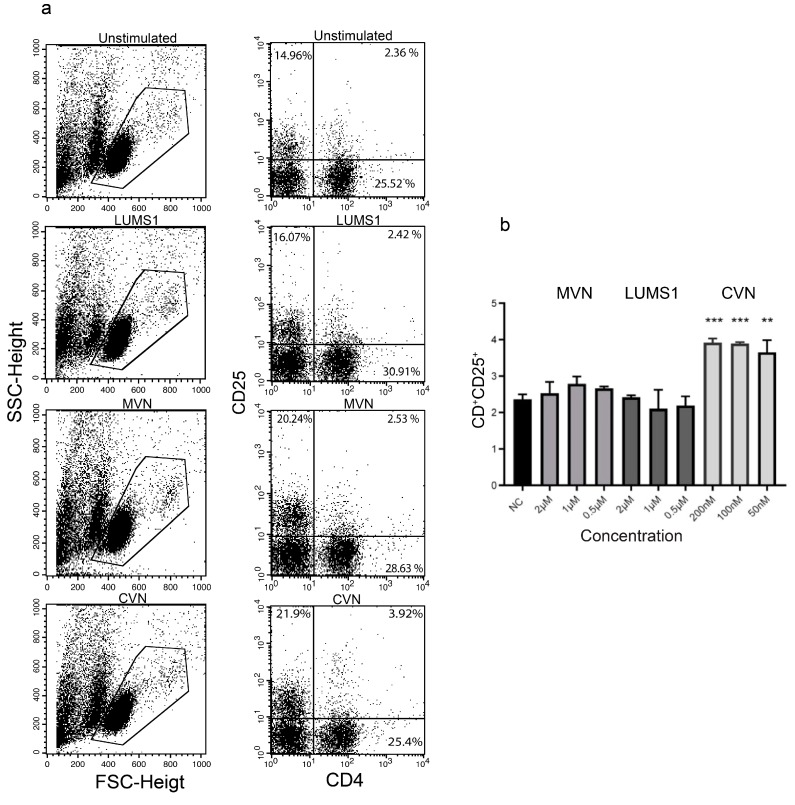
Effect of LUMS1 on the activation of T_h_ cells: (**a**) Flow cytometry analysis of PBMCs to determine the population of CD4^+^ and CD25^+^ cells in freshly isolated PBMCs in response to treatment with LUMS1, MVN, cyanovirin-N (CVN). After treating PBMCs (10^6^ cells/mL) with varying concentrations of LUMS1, MVN, and CVN for 72 h at 37 °C and 5% CO_2_, cells were washed with PBS and incubated with APC-conjugated anti-CD4 and PE-conjugated anti-CD25 antibodies for 30 min at 4 °C. Finally, cells were washed with PBS (2% FBS), fixed with 1% formaldehyde, and analyzed by FACS, using CellQuest software for data acquisition. Data were statistically analyzed using GraphPad Prism software. (**a**) Left panel, representative dot plots of forward scatter (FSC) and side scatter (SSC) indicating the subpopulation of cells in PBMCs; right panel, dot plots showing the relative population of cells with CD4 and CD25 activation markers. (**b**) Plot showing the percent population of CD4^+^ and CD25^+^ cells after treating with LUMS1, MVN, and CVN separately. The data represent the mean of three independent experiments and one-way ANOVA was used to compare different groups. ** *p* ≤ 0.01; *** *p* ≤ 0.001.

In the context of evaluating immunogenic effects of a protein in vitro, activation of T_h_ and B cells is considered an important marker of immunogenicity as these cells are involved in inducing monoclonal antibody-based immunogenicity [26,27]. In this regard the effect of LUMS1 and in parallel of MVN, was analyzed on the expression of CD4, CD25, and CD20 activation markers through flow cytometry (FACS) using freshly isolated PBMCs from healthy individuals. CVN was used as a positive control for the expression of CD4 and CD25 as its effect on these cellular activation markers have already been reported [28]. Both LUMS1 and MVN did not increase the population of CD4^+^ and CD25^+^ cells (T_h_ cells), while CVN in this case showed significantly high activation even at a concentration of 50 nM (Figure 5).

**Figure 6:**
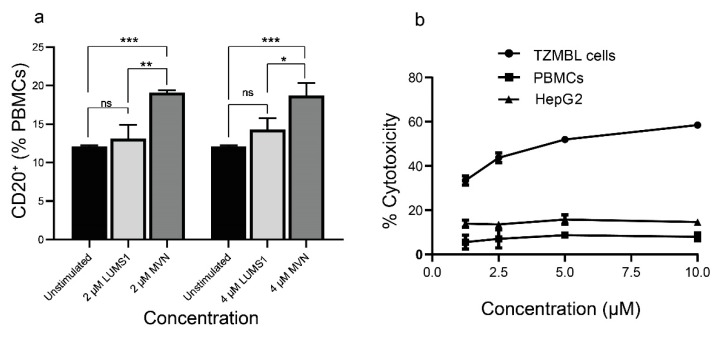
Evaluation of B cells activation by LUMS1 and its cytotoxicity: (**a**) graph presenting the flow cytometry analysis to determine the population of CD20^+^ cells in freshly isolated PBMCs. PBMCs were isolated from freshly collected blood using density gradient centrifugation and washed with PBS. After treating PBMCs (10^6^ cells/mL) with varying concentrations of LUMS1 and MVN for 72 h at 37 °C and 5% CO_2_, cells were washed with PBS and incubated with percp cy5.5-conjugated anti-CD20 antibodies for 30 min at 4 °C. Finally, cells were washed with PBS (2% FBS), fixed with 1% formaldehyde and analyzed by FACS, using CellQuest software for data acquisition. Data were statistically analyzed using GraphPad Prism software. Analyses were performed in triplicate and one-way ANOVA with multiple comparisons was used to compare different groups. * *p* ≤ 0.05, ** *p* ≤ 0.01; *** *p* ≤ 0.001. (**b**) The plot showing concentration dependent cytotoxic effect of LUMS1 on PBMCs, HepG2, and TZM-bl cells.

However, LUMS1 showed more a pronounced difference with MVN on the activation of CD20^+^ cells. Treatment of LUMS1 at a concentration as high as 4 μM did not significantly increase the population of CD20^+^ cells, while the effect of MVN in this regard was significantly high even at 2 μM concentration (Figure 6a). In addition to evaluating the cytotoxicity of LUMS1 against Huh7.5 cells (Figure 4a,c) we also determined the effect of LUMS1 on the viability of TZM-bl cells, PBMCs, and HepG2 cells using MTT assay. LUMS1 did not show a cytotoxic effect on Huh7.5 cells, HepG2 cells, and PBMCs at a concentration as high as 10 µM whereas its CC_50_ value against TZM-bl cells was calculated as 4.9 ± 0.166 µM (Figure 6b and Table 2).

## 4. Discussion

In this study, we engineered a lectin, LUMS1, by modifying MVN to incorporate two carbohydrate-binding sites and reduce chemical heterogeneity, a major factor in potential immunogenicity of a protein. The NMR analysis of the carbohydrate binding of LUMS1 suggested that it exhibited two carbohydrate-binding sites, and it has the same carbohydrate specificity as MVN—both recognize the α(1-2)mannobiose glycan as the minimum epitope.

MVN has been reported to inhibit HIV-1 entry with EC_50_ values ranging from 2 to 12 nM against HIV-1, and we reproduced the reported EC_50_ against HXB2 strain of HIV-1. LUMS1, however, inhibited the same strain with ≈4.5-fold lower potency (EC_50_ 37 nM). LUMS1 contains two carbohydrate-binding sites and is expected to exhibit higher avidity as compared to MVN by engaging more than one glycan or glycan branches at the surface of the virus. Lower potency of LUMS1 against HIV-1 as compared to MVN could be attributed to the different possible mechanism by which these lectins attain high avidity of interactions with the viral envelope; multivalent recognition in the case of LUMS1 and the bind-and-hop mechanism through single site interactions in the case of MVN [19], although these mechanisms remain to be experimentally validated. Binding studies of these lectins with the stabilized HIV-1 gp120 trimer, the form of the envelope protein exists on the viral spike, through isothermal titration calorimetry (ITC) or fluorescence resonance energy transfer (FRET) measuring microevents of binding could illustrate the detailed mechanism [29,30]. Moreover, multivalent recognition can be identified by solving the structure of the complex of lectin and gp120-trimer through X-ray or cryo electron microscopy. While comparing the carbohydrate binding of these lectins, the cross-peaks of carbohydrate-binding site amino acids of MVN in ^1^H-^15^N correlation NMR spectra experienced chemical shift changes on the addition of ligand suggesting slow exchange on the NMR time scale [19]. On the other hand, in the case of LUMS1, chemical shift perturbation in most of the cross-peaks were in the form of line broadening suggesting intermediate exchange on the NMR time scale. Slow exchange on the NMR time scale is related to higher binding affinity (lower K_D_ values) as compared to intermediate exchange [31]. The two lectins, therefore, demonstrate the difference in binding to carbohydrate in terms of affinity. The apparent lower carbohydrate-binding affinity of LUMS1 as compared to MVN indicates that its carbohydrate-binding sites may not be structurally optimal, which could be understood only after the structure of the complex of LUMS1 and carbohydrate is available. However, one of the significant aspects of LUMS1 was found to be its ability to potently inhibit HCV infection with an EC_50_ of 45 and 142.1 nM as determined in HCVcc and HCVpp assays, respectively. HCV inhibition by different oligomeric forms of MVN has been reported but only qualitatively [32]. By testing LUMS1 in HCVpp and replicon system, in addition to HCVcc assay, we clearly demonstrated that exclusively HCV E1/E2-mediated viral entry was inhibited. Many HIV-1 entry inhibitor lectins have been reported also to inhibit the entry of HCV [33,34,35], which could be attributed to likely similar glycan density on the surface of both viruses [8,36]. This apparent similarity potentiates the development of universal therapy against both viruses. Moreover, LUMS1 demonstrated its specificity for HIV-1 and HCV, as it did not inhibit an amphotropic virus VSV.

The major obstacle in the advancement towards clinical application of anti-viral lectins is their potential cytotoxicity and immunogenicity [17,27]. In cell viability assays using four different types of cells, LUMS1 demonstrated negligible cytotoxic effects with CC_50_ > 10 µM against Huh7.5 cells, HepG2 cells and PBMCs, and 4.9 ± 0.166 μM against the TZM-bl cells with a selectivity index (SI) value of 108, calculated by the ratio of the smallest CC_50_ value (4.9 µM) and the EC_50_ value (45.3 nM, against HCVcc) indicating its promising safety profile. As foreign peptides are prone to induce immunogenicity in patients, we tested LUMS1 for its effect on the activation of B and T_h_ cells in vitro and observed that LUMS1 demonstrated no significant increase in the expression of activation markers for these cells at a concentration as high as 4 μM. LUMS1 demonstrated significantly lesser effect in inducing the CD20 activation marker however its effect on the induction of CD4 and CD25 activation markers was comparable to MVN but slightly less. The detailed safety profile of LUMS1, however, remains to be investigated in animal models in the follow-up study. Taken together, LUMS1 represents an attractive potential therapeutic candidate against HIV-1 and HCV, as it potently inhibits both of these viruses, demonstrates lack of cytotoxicity and negligible activation of B and T_h_ cells. With the emerging trend of protein drugs, further optimization of LUMS1 to enhance its carbohydrate-binding affinity leading to increase anti-viral potency, and its detailed investigation in vitro and in vivo are the further aspects to be considered.

## Figures and Tables

**Table 1:** Overview of antiviral activity of LUMS1 and compounds used as positive control.

Assay System	Inhibitor	EC_50_ (nM)
HCVcc	LUMS1	45.3 ± 18.6
	MVN ^#^	35.6 ± 3.98
HCV replicon	LUMS1	n.d.
	Sofosbuvir	54.0 ± 32.8
HCVpp	LUMS1	142.1 ± 23.0
HIV-1 single round infectivity	LUMS1	37.2 ± 4.4
	MVN	8.0 ± 1.4

^#^ Dose response curve is given in Figure S3, Supplementary Material.

**Table 2:** Overview of the cytotoxicity of LUMS1 against different cell lines.

Cell Types	CC_50_ of LUMS1 (nM)
Huh7.5	>10,000
PBMCs	>10,000
TZMbl	4900 ± 166
HepG2	>10,000

## Supplementary Materials

The following are available online at https://www.mdpi.com/1999-4915/12/2/199/s1, Detailed methods about protein expression; Figure S1, Image containing overlaid structures of MVN and LUMS1; Figure S2, Effect of LUMS1 on VSV infection; and Figure S3, Anti-HCV activity of MVN.

## References

[1] Jefferies M., Rauff B., Rashid H., Lam T., Rafiq S. (2018). Update on global epidemiology of viral hepatitis and preventive strategies. World J. Clin. Cases.

[2] Mahy M., Marsh K., Sabin K., Wanyeki I., Daher J., Ghys P.D. HIV estimates through 2018: Data for decision making. Aids.

[3] Ashraf M.U., Iman K., Khalid M.F., Salman H.M., Shafi T., Rafi M., Javaid N., Hussain R., Ahmad F., Shahzad-Ul-Hussan S. (2019). Evolution of efficacious pangenotypic hepatitis C virus therapies. Med. Res. Rev..

[4] Dhiman R.K., Grover G.S., Premkumar M. Hepatitis C elimination: A Public Health Perspective. Curr. Treat. Options Gastroenterol..

[5] Kanters S., Vitoria M., Doherty M., Socias M.E., Ford N., Forrest J.I., Popoff E., Bansback N., Nsanzimana S., Thorlund K. (2016). Comparative efficacy and safety of first-line antiretroviral therapy for the treatment of HIV infection: A systematic review and network meta-analysis. Lancet HIV.

[6] Goffard A., Dubuisson J. (2003). Glycosylation of hepatitis C virus envelope proteins. Biochimie.

[7] Doores K.J., Bonomelli C., Harvey D.J., Vasiljevic S., Dwek R.A., Burton D.R., Crispin M., Scanlan C.N. (2010). Envelope glycans of immunodeficiency virions are almost entirely oligomannose antigens. Proc. Natl. Acad. Sci. USA.

[8] Fenouillet E., Gluckman J.C., Jones I.M. (1994). Functions of HIV envelope glycans. Trends Biochem. Sci..

[9] Shahzad-ul-Hussan S., Ghirlando R., Dogo-Isonagie C.I., Igarashi Y., Balzarini J., Bewley C.A. (2012). Characterization and carbohydrate specificity of pradimicin S. J. Am. Chem. Soc..

[10] Balzarini J. (2007). Carbohydrate-binding agents: A potential future cornerstone for the chemotherapy of enveloped viruses?. Antivir. Chem. Chemother..

[11] Mori T., O’Keefe B.R., Sowder R.C., Bringans S., Gardella R., Berg S., Cochran P., Turpin J.A., Buckheit R.W., McMahon J.B. (2005). Isolation and characterization of griffithsin, a novel HIV-inactivating protein, from the red alga Griffithsia sp.. J. Biol. Chem..

[12] Akkouh O., Ng T.B., Singh S.S., Yin C., Dan X., Chan Y.S., Pan W., Cheung R.C. (2015). Lectins with anti-HIV activity: A review. Molecules.

[13] Yang H., Li J., Patel S.K. (2019). Design of Poly(lactic-co-glycolic Acid) (PLGA) Nanoparticles for Vaginal Co-Delivery of Griffithsin and Dapivirine and Their Synergistic Effect for HIV Prophylaxis. Pharmaceutics.

[14] O’Keefe B.R., Vojdani F., Buffa V., Shattock R.J., Montefiori D.C., Bakke J., Mirsalis J., d’Andrea A.L., Hume S.D., Bratcher B. (2009). Scaleable manufacture of HIV-1 entry inhibitor griffithsin and validation of its safety and efficacy as a topical microbicide component. Proc. Natl. Acad. Sci. USA.

[15] Huskens D., Schols D. (2012). Algal lectins as potential HIV microbicide candidates. Mar. Drugs.

[16] Griffithsin-Based Rectal Microbicide for PREvention of Viral ENTry (PREVENT). https://clinicaltrials.gov/ct2/show/NCT04032717.

[17] Kehr J.C., Zilliges Y., Springer A., Disney M.D., Ratner D.D., Bouchier C., Seeberger P.H., de Marsac N.T., Dittmann E. (2006). A mannan binding lectin is involved in cell-cell attachment in a toxic strain of Microcystis aeruginosa. Mol. Microbiol..

[18] Huskens D., Ferir G., Vermeire K., Kehr J.C., Balzarini J., Dittmann E., Schols D. (2010). Microvirin, a novel alpha(1,2)-mannose-specific lectin isolated from Microcystis aeruginosa, has anti-HIV-1 activity comparable with that of cyanovirin-N but a much higher safety profile. J. Biol. Chem..

[19] Shahzad-ul-Hussan S., Gustchina E., Ghirlando R., Clore G.M., Bewley C.A. (2011). Solution structure of the monovalent lectin microvirin in complex with Man(alpha)(1-2)Man provides a basis for anti-HIV activity with low toxicity. J. Biol. Chem..

[20] Gustchina E., Louis J.M., Lam S.N., Bewley C.A., Clore G.M. (2007). A monoclonal Fab derived from a human nonimmune phage library reveals a new epitope on gp41 and neutralizes diverse human immunodeficiency virus type 1 strains. J. Virol..

[21] Lee M., Yang J., Park S., Jo E., Kim H.Y., Bae Y.S., Windisch M.P. (2016). Micrococcin P1, a naturally occurring macrocyclic peptide inhibiting hepatitis C virus entry in a pan-genotypic manner. Antivir. Res..

[22] Hsu M., Zhang J., Flint M., Logvinoff C., Cheng-Mayer C., Rice C.M., McKeating J.A. (2003). Hepatitis C virus glycoproteins mediate pH-dependent cell entry of pseudotyped retroviral particles. Proc. Natl. Acad. Sci. USA.

[23] Baldick C.J., Wichroski M.J., Pendri A., Walsh A.W., Fang J., Mazzucco C.E., Pokornowski K.A., Rose R.E., Eggers B.J., Hsu M. (2010). A novel small molecule inhibitor of hepatitis C virus entry. PLoS Pathog..

[24] Kumar P., Nagarajan A., Uchil P.D. (2018). Analysis of cell viability by the MTT assay. Cold Spring Harb. Protoc..

[25] Bewley C.A., Shahzad-Ul-Hussan S. (2013). Characterizing carbohydrate-protein interactions by nuclear magnetic resonance spectroscopy. Biopolymers.

[26] Baker M.P., Reynolds H.M., Lumicisi B., Bryson C.J. (2010). Immunogenicity of protein therapeutics: The key causes, consequences and challenges. Self Nonself.

[27] Ito S., Ikuno T., Mishima M., Yano M., Hara T., Kuramochi T., Sampei Z., Wakabayashi T. (2019). In vitro human helper T-cell assay to screen antibody drug candidates for immunogenicity. J. Immunotoxicol..

[28] Huskens D., Vermeire K., Vandemeulebroucke E., Balzarini J., Schols D. (2008). Safety concerns for the potential use of cyanovirin-N as a microbicidal anti-HIV agent. Int. J. Biochem. Cell. Biol..

[29] Lee J.H., Ozorowski G., Ward A.B. (2016). Cryo-EM structure of a native, fully glycosylated, cleaved HIV-1 envelope trimer. Science.

[30] Dam T.K., Brewer C.F. (2008). Effects of clustered epitopes in multivalent ligand-receptor interactions. Biochemistry.

[31] Li Y., Kang C. (2017). Solution NMR Spectroscopy in Target-Based Drug Discovery. Molecules.

[32] Min Y.Q., Duan X.C., Zhou Y.D., Kulinich A., Meng W., Cai Z.P., Ma H.Y., Liu L., Zhang X.L., Voglmeir J. (2017). Effects of microvirin monomers and oligomers on hepatitis C virus. Biosci. Rep..

[33] Takebe Y., Saucedo C.J., Lund G., Uenishi R., Hase S., Tsuchiura T., Kneteman N., Ramessar K., Tyrrell D.L., Shirakura M. (2013). Antiviral lectins from red and blue-green algae show potent in vitro and in vivo activity against hepatitis C virus. PLoS ONE.

[34] Kachko A., Loesgen S., Shahzad-Ul-Hussan S., Tan W., Zubkova I., Takeda K., Wells F., Rubin S., Bewley C.A., Major M.E. (2013). Inhibition of hepatitis C virus by the cyanobacterial protein Microcystis viridis lectin: Mechanistic differences between the high-mannose specific lectins MVL, CV-N, and GNA. Mol. Pharm..

[35] Helle F., Wychowski C., Vu-Dac N., Gustafson K.R., Voisset C., Dubuisson J. (2006). Cyanovirin-N inhibits hepatitis C virus entry by binding to envelope protein glycans. J. Biol. Chem..

[36] Goffard A., Callens N., Bartosch B., Wychowski C., Cosset F.L., Montpellier C., Dubuisson J. (2005). Role of N-linked glycans in the functions of hepatitis C virus envelope glycoproteins. J. Virol..

