# Supplementary material for: An Engineered Microvirin Variant with Identical Structural Domains Potently Inhibits Human Immunodeficiency Virus and Hepatitis C Virus Cellular Entry
Source: Viruses. 2020 Feb 11;12(2):199. doi: 10.3390/v12020199 (PMC7077325; doi:10.3390/v12020199)
Supplement: Supplementary file 1 [file viruses-12-00199-s001.pdf]

Supplement Material

Protein expression

For the recombinant expression of LUMS1, the DNA sequence encoding for LUMS1 amino acid sequence and enterokinase cleavage site just before the LUMS1 gene, was synthesized through commercial facilities (Genscript, USA). The construct contained *Bam*H I and *Xho* I restriction sites at 5′ and 3′ ends, respectively. By using these restriction enzymes the gene was sub-cloned into pET32a expression vector. The sub-cloning was verified through agarose gel electrophoresis and commercial DNA sequencing (Macrogen Inc. Korea). The plasmid containing the required gene was transformed into BL21 strain of Escherichia coli, which was grown at 37°C in LB media, induced with 1 mM IPTG at OD_{600} of 0.6 and harvested four hours after the induction. After lysis the supernatant was subjected to nickel-affinity chromatography to purify the expressed protein which also contained histidine and thioredoxin tags. The purified protein was treated with enterokinase enzyme to cleave off the tags and the LUMS1 protein was purified through size exclusion followed by ion exchange chromatography. For the expression of ^{15}N-labelled protein, the transformed bacteria were grown in minimal media supplemented with ^{15}N-ammonium chloride as the only source of nitrogen. The purified protein was transferred into PBS buffer of pH 7.4 for all biological assays, and into 20 mM phosphate buffer containing 50 mM NaCl for NMR experiments, through dialyses using dialysis membrane of 3.5 KDa cutoff (Slide-A-Lyzer™ MINI Dialysis Device, Thermo Fisher, USA) [1].

References

1. Shahzad-ul-Hussan, S.; Gustchina, E.; Ghirlando, R.; Clore, G.M.; Bewley, C.A. Solution structure of the monovalent lectin microvirin in complex with Man(alpha)(1-2)Man provides a basis for anti-HIV activity with low toxicity. *J Biol Chem* **2011**, *286*, 20788-20796, doi:10.1074/jbc.M111.232678.

Supplementary Figures

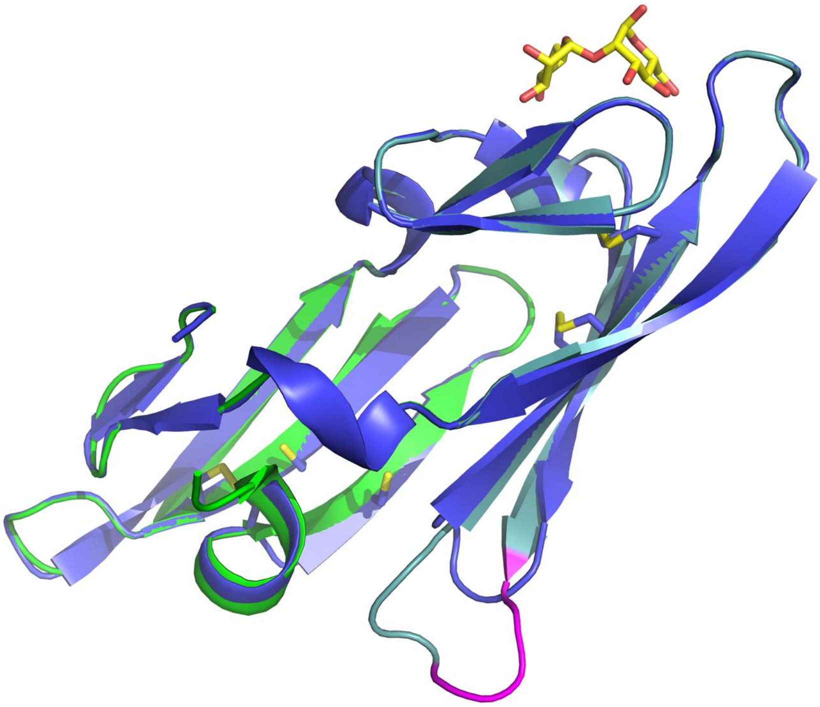

Figure S1. Cartoon presentation of the overlaid structures of MVN and LUMS1. LUMS1 is colored blue while domains -A and -B of MVN are colored cyan and bright green, respectively. MVN bound glycan and side chains of all cysteine residues of both proteins are colored yellow. Four-residues of MVN domain-A which are not included in LUMS1 are colored pink.

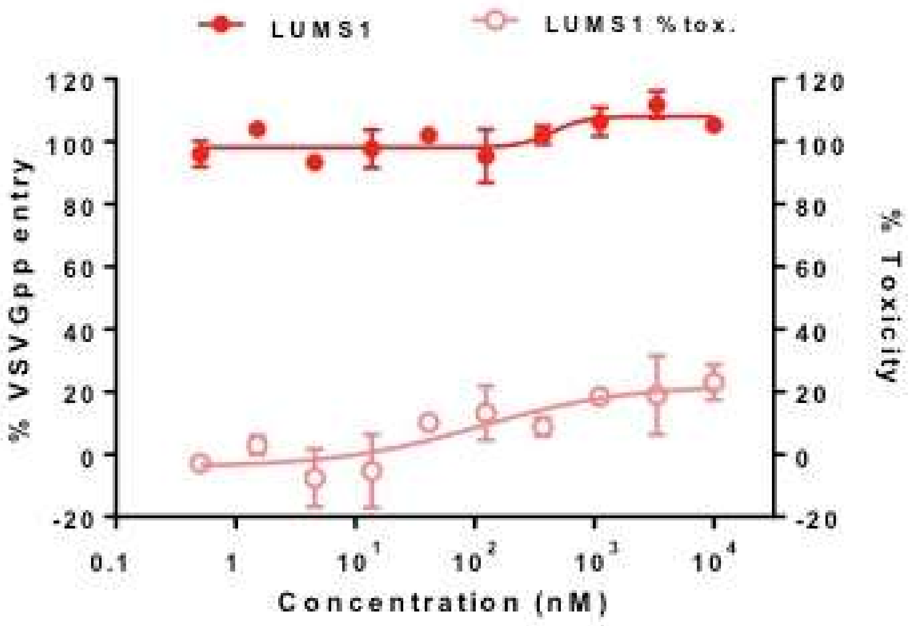

Figure S2. Effect of LUMS1 on VSV infection: A dose response curve showing the effect of LUMS1 on the infection of VSV.

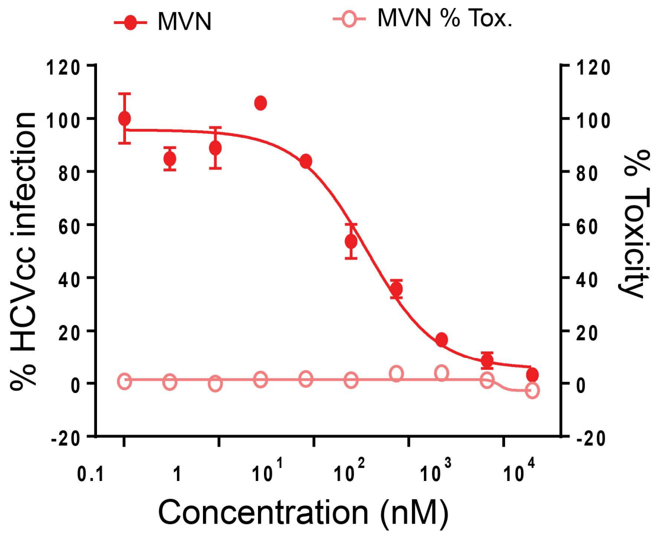

Figure S3. Anti-HCV activity of MVN. Huh-7.5 cells were pretreated with increasing concentrations of MVN for 2h followed by infection with HCVcc (JFH1) for 72h in the presence of proteins. (B) HCV infectivity was assessed by calculating the number of GFP-positive cells for 3 days in the presence of the protein. EC_{50} of MVN was determined as 35.65 ± 3.98 nM. Cytotoxicity of MVN was assessed by counting cell nuclei stained with Hoechst 33342 and normalized to untreated control cells.
